# Translating Coverage Gains into Health Gains for All Women and Children: The Quality Care Opportunity

**DOI:** 10.1371/journal.pmed.1001368

**Published:** 2013-01-15

**Authors:** Wendy J. Graham, Affette McCaw-Binns, Stephen Munjanja

**Affiliations:** 1 Immpact, School of Medicine and Dentistry, University of Aberdeen, Aberdeen, United Kingdom; 2 Department of Community Health and Psychiatry, University of West Indies-Mona, Kingston, Jamaica; 3 Department of Obstetrics and Gynaecology, University of Zimbabwe, Harare, Zimbabwe

## Abstract

Wendy Graham and colleagues reflect on quality of maternal health care, the focus of Year 1 of the PLOS-MHTF Maternal Health Collection and its 18 new articles.

**Linked Collection**

This Perspective was commissioned to reflect on the *quality of maternal health care*, the theme of Year 1 of the Maternal Health Task Force-PLOS Collection on maternal health.

The Year 1 Collection contains 18 new, outstanding research and commentary articles from a wide range of settings and authors in developed and developing countries, providing needed evidence to improve the quality of maternal health care worldwide.

More information and the full list of articles can be found at the Collection page: http://www.ploscollections.org/maternalhealth

The proportion of women and children receiving health care in the poorest countries is increasing [1]. Unfortunately, markers of improved health outcomes, such as falling maternal or newborn mortality, have not matched expectations from the gains in the coverage of care. Robust evidence exists for one explanatory factor: the poor–rich gaps in coverage found along the continuum of care for women and children, and particularly for the crucial period around childbirth [2]. The more-neglected explanation for the mismatch between coverage and health outcomes is the quality of the care provided to women and children, which is the focus of Year 1 of the Maternal Health Task Force (MHTF)-PLOS Collection on Maternal Health (http://www.ploscollections.org/maternalhealth_year1) and our short commentary.

Although inadequacies in care have long been noted across the world and for many health problems [3], a focus on the magnitude, costs, and consequences specifically for women and children in low-income countries is relatively new, and still has not achieved the status of a political priority [4]. This contrasts markedly with the attention paid to the coverage of care. Here we seek to highlight the synergies between inequalities in coverage *and* quality. The inverse care law [5] proposes that quality of care varies inversely with need, and we extend this to emphasize that poor quality care is disproportionately borne by the poorest groups of women and children. Our commentary is structured around a key cause and a consequence of the neglect of quality—weak measurement and poor evidence for action—and concludes with priorities for seizing the quality care opportunity.

## Measurement Traps

In global health there are numerous examples of measurement constraints distorting priorities for policies, programmes, and research [6]. In maternal health, this situation has been described as a *measurement trap* [7] that reflects both weak routine information systems and a lack of consensus on critical concepts, definitions, and tools. The diversity of interpretations of what constitutes *quality* and thus *quality care* can be seen across the 18 articles in this MHTF-PLOS Collection. In our commentary we adopt the well-accepted definition: “quality means clinical effectiveness, safety, and a good experience for the patient" [8] and give three examples of how measurement traps impact on the evidence and understanding of quality.

The weak routine patient record systems in many low-income countries present serious challenges to quality assessment and improvement, using tools such as criterion-based audit, to medical liability, and crucially, to clinical case management. Selection biases in the cases reviewed arise from incomplete and inaccurate registers, and conclusions on standards of clinical care may be distorted by missing or poorly completed patient records [9]. The underreporting of adverse outcomes, such as maternal deaths [10], can undermine local recognition of poor quality care and the need for corrective action. Measurement and data constraints have, in turn, led to a failure to recognise levels of quality along a continuum, and to neglect of essential requirements for delivering quality care, like basic infrastructure for providing water and sanitation in facilities [11],[12].

A second measurement trap relates to the data source for most coverage indicators—cross-sectional population surveys. The Demographic and Health Surveys (DHS) have made an enormous contribution in low-income countries, providing a picture of coverage based on women's own reports of health care services or interventions that they or their children needed and received. However, not all sub-groups or types of experiences of care are represented. One such group is women experiencing stillbirths, since the majority of the questions in the DHS relate to women with live births. These surveys are used to judge country progress in coverage [1], and thus population biases are relevant. Studies have found that women with live births are more likely than those with stillbirths to tolerate poor quality care [13] and to feel less need for skilled care in subsequent pregnancies [14]. The well-established link between adverse birth events and inadequate care, and the clustering of both poor outcomes and poor care among the poorest women, emphasizes the urgent need for co-measurement of coverage *and* quality.

The third example comes from the dominance of *supply-side* approaches to routinely measuring quality in low-income settings. Functioning health systems that have successfully embedded a culture of quality respond to the *demand-side* by continuously capturing the views and voices of users, both in private and in public service provision [15]. This Collection and other articles offer valuable insights from research studies on women's expressed needs, such as trust and respect during childbirth, and there are novel techniques, like photo-narratives [16], for capturing these views. However, studies typically have special skills and resources at their disposal, and thus tools developed through research [17] may not be feasible for routine quality assessment, improvement, and assurance in low-income countries.

## Evidence Gaps

Quality care can be viewed as either a desired goal, and so used to judge the health system, or as an intervention for achieving health outcomes, such as improved survival. In both cases, the major evidence gap is *how* to implement care that is clinically effective, safe, and a good experience for the patient. Quality care is thus part of the wider challenge of implementation in global health, characterised by a weak evidence base and underdeveloped science [18]. Not only are there technical issues to delivering quality care, but also social and cultural factors, making the implementation context crucial [19]. Relevant evidence and lessons will thus emerge not solely from research, and ways to capture and synthesise programmatic experience are also needed.

To identify priority evidence gaps in the implementation of quality care for women and children, we recommend a consensus-building exercise at country or regional levels [20]. Here we express our own views on just three key gaps. Firstly, there is a notable lack of evidence on financial issues in relation to quality. This includes robust costing of quality improvement and assurance interventions, as well as the impact of financing mechanisms, such as pay for performance, on quality in both public and private contexts. Improving quality is widely viewed as a net cost, and more evidence is needed on financial savings in addition to lives saved [21]. The second evidence gap relates to the strong behavioural component of quality improvement among providers and women users, and the need for deeper insights to help explain attitudes and practises, such as motivation and conscientiousness. Inputs should be sought from a wider range of disciplines, including psychology and organisational science, as well as from other sectors, like commerce, for the management of performance and risks. Finally, we highlight the evidence gaps on patient safety strategies and interventions. The basic principle of “first do no harm" has particular resonance in maternity care [22]. The mounting evidence of high levels of harm in health care institutions in low-income countries suggests the need for a systems approach to human error, such as James Reason's [23] “Swiss cheese model," which shows how the alignment of failings in health system safeguards brings hazards into contact with victims. There are examples of such alignment and of novel responses in this Collection, including Spector and colleagues' [24] checklist approach to ensuring patient safety.

## Seizing the Quality Care Opportunity

Improving and assuring the quality of care received by all women and children in low-income countries is crucial to achieving health, equity, and human rights goals. Now is the time to seize this opportunity and to reposition quality at the centre of debates on universal health coverage and in post-2015 development priorities. “Effective coverage" should be the new narrative in these debates, meaning high and equitable coverage of quality care. This repositioning requires a fundamental shift in policy and programme mindsets to accept quality care as essential to protecting lives, well-being, and scarce health resources. We conclude by highlighting four priorities to help create a policy, programme, and research environment conducive to quality health systems.

1. **Listen to women users**
  Quality health systems are those that respond to the people they serve. The challenges of capturing the valid views and opinions of women users of public and private services in low-income countries are non-trivial, particularly outside of a research context. Tackling these will require commitment from policy-makers, managers, and practitioners, and should be linked to the development of accountability frameworks. Although these frameworks have often focused on community voices around adverse events like maternal deaths, this needs to broaden to include the perspectives of women survivors with good and bad care experiences.
2. **Create a learning movement**
  Assuring quality care for women and children in low-income settings is fundamentally about implementation. A learning movement is needed to enable efficient exchange of implementation lessons, and to organise these in terms of contexts. Some, but not all, lessons will be research-based, and disseminating these will require funders and journals to temper their biases towards only reporting success, since it is equally important to know what doesn't work. Such an honest and open learning movement needs to be multi-disciplinary, multi-professional, and multi-sectoral, with a willingness to think and work outside the box, to capture innovative thinking from the global south and north and from business and industry, and to be action-focused. The next generation of service providers must also be nurtured by the movement and supported by a system that enables woman-centred care, best practices, and continuing professional development. The learning and implementation experiences of all care providers must be voiced to policy decision-makers and to civil society.
3. **Get back to basics**
  Focusing on the human dimension to quality health systems has been understandable, but the basic physical environment in which providers practice and women and children receive care needs comparable attention. The poor state of water and sanitation provision in maternity units, for instance, is alarming and, along with poor hygiene practices, presents a real risk of harm and a return to epidemics of infection, complicated by the 21st century problem of antibiotic resistance. There is a need to routinely audit adequacy of basic infrastructure and equipment, to link this performance to accountability frameworks, and to ensure relevant indicators are included in post-2015 monitoring platforms.
4. **Invest in patient records and registers**
  The very foundations of quality assessment and assurance processes—patient records and registers—remain inadequate in many low-income countries, and often have not benefited in broader efforts to strengthen health information systems. The situation must change whereby women and children entering the health care system remain invisible since patient records are not issued nor entries made in registers. Such records and registers should be seen as fundamental to case management and clinical training, and to detecting important changes in health burdens so the service can respond. Innovations are needed in capture, collation, and storage, such as simple electronic registers and woman-held case notes, and in disaggregating data to enable routine assessment of the quality of care received by the most disadvantaged women and children. These improvements must extend through to the use of data for action, from the clinical setting to policy levels, and these need strong political and financial investment. The state of patient records and registers is ultimately a mirror of quality health systems and thus also a valid development target.

We have focused on quality care specifically for women and children. But seizing the quality care opportunity will also benefit the wider health system, care providers, and other population groups.

## Abbreviations

DHS: Demographic and Health Surveys
MHTF: Maternal Health Task Force
